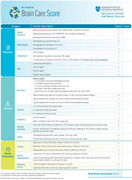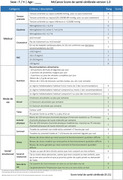# Adapting and validating the McCance Brain Care Score for French‐speaking patients: a prospective study on motivation to improve Brain Health in a neurology outpatient clinic

**DOI:** 10.1002/alz70860_104053

**Published:** 2025-12-23

**Authors:** Hadrien M Lalive, Arthur J. L. Watkins, Mathilde d'Esneval, Alexandra Rhally, Sophie Bernasconi‐Xhepa, Alma Lingenberg, Jasper Senff, Sanjula D Singh, Frédéric Assal, Jonathan Rosand, Lukas Sveikata

**Affiliations:** ^1^ Division of Neurology, Geneva University Hospitals, Geneva, Switzerland; ^2^ Department of Neurology, Massachusetts General Hospital, Boston, MA, USA

## Abstract

**Background:**

Promoting brain health is essential for addressing the growing burden of neurological disorders, with 45% of dementia cases linked to modifiable risk factors. The McCance Brain Care Score (BCS, Figure 1) supports risk reduction by empowering patients to adopt preventive strategies that modify established risk factors. However, no validated French‐language tools currently address modifiable determinants of brain health. This prospective study aims to validate a French version of the BCS (BCS‐F, Figure 2) and evaluate its effectiveness in motivating patients to improve their brain health.

**Method:**

The BCS was translated into French through a forward and back translation process conducted by two independent translators, followed by validation by an expert committee. 95 patients (mean age 70.7±13.4 years, 39% female, mean MoCA score 22.3±5.5/30 points) were recruited from the Cognitive Disorders Outpatient Clinic between March 2024 and January 2025. 43 consecutive participants were assigned to the BCS‐F group and 52 to the standard of care (SoC) group. Key outcomes included patients’ willingness to improve brain health determinants and items targeted for change, which were compared between groups.

**Result:**

Both groups were comparable in age, sex and general cognitive performance. 81 % (*n* = 35) of participants in the BCS‐F group and 48% (*n* = 25) of participants in the SoC group expressed a willingness to improve their brain health determinants, with blood pressure control, aerobic activity and alcohol consumption being the most frequently targeted factors. The administration of the BCS‐F was associated with a 69% increased probability of willingness to improve brain health (Risk Ratio: 1.69, *p* <0.001) compared to SoC, even after accounting for age as a confounding variable. One additional participant reported willingness to improve their brain health for every 3 patients who completed the survey (Number needed to treat: 3.00).

**Conclusion:**

The BCS‐F proved an effective intervention in our cohort, enhancing willingness to act on brain health determinants. This suggests that BCS‐F has the potential to address the scarcity of accessible brain health assessment tools for French‐speaking populations. Furthermore, BCS‐F may serve as a valuable resource for informing patients about their brain health and empowering them to improve modifiable risk factors for dementia, stroke, and depression.